# Neurostimulation as an Efficacious Nonpharmacologic Analgesic following Arthroscopic Rotator Cuff Repair

**DOI:** 10.1155/2022/2133998

**Published:** 2022-04-15

**Authors:** Ryan B. Juncker, Joel J. Gagnier, Faisal M. Mirza

**Affiliations:** ^1^Department of Orthopaedic Surgery, David Geffen School of Medicine, University of California, Los Angeles (UCLA), Los Angeles, CA, USA; ^2^Department of Orthopaedic Surgery, Department of Epidemiology, University of Michigan, Ann Arbor, MI, USA; ^3^Coastal Health Partners, Watsonville, CA, USA

## Abstract

This case highlights the importance of pursuing nonpharmacologic analgesic modalities in orthopedic surgery to combat the current opioid epidemic. Presented is a patient who underwent an arthroscopic rotator cuff repair and biceps tenodesis operation and through the use of neurostimulation (in the form of auricular electrostimulation), fully recovered from surgery without the usage of any opioid or nonsteroidal anti-inflammatory medications. The patient was fitted with a novel auricular electrostimulation device (DyAnsys Primary Relief) in the immediate postoperative period that provided constant neurostimulation for 10 days, this neurostimulator was the only analgesic modality used in this case, and the patient reported minimal postoperative pain. The utility of this case centers around the lack of postoperative opioid use, presenting the idea that postsurgical orthopedic pain can be managed in a nonpharmacologic capacity, combatting the fields' ongoing opioid epidemic.

## 1. Introduction

Degeneration of the rotator cuff is a common issue treated by orthopedic surgeons. A complete rotator cuff tear has been shown as the third most common cause of musculoskeletal complaints to physicians and constitutes 30–70% of reported shoulder pain [1–3]. Furthermore, rotator cuff disease has been shown in multiple studies to be prevalent in 22–32% of the population aged sixty and over [4, 5]. Surgical intervention is considered an optimal practice for the full-thickness complete rotator cuff tears that are refractory to nonoperative care [1]. In addition to arthroscopic rotator cuff repair, biceps tenodesis operations are commonly performed by orthopedic surgeons to treat tendinopathy of the long head of the biceps identified through fraying, flattening, or lesions of the biceps tendon [6, 7]. Recent research has shown operative management to be successful in pain relief and functional restoration of the biceps tendon and shoulder joint [6].

Based on a survey conducted in 2018 of American Shoulder and Elbow Surgeons members and two recently conducted systematic reviews, common practice peri and postoperative anesthesia/analgesia for rotator cuff repair operations consists of a nerve block(s), prescription of nonsteroidal anti-inflammatory drugs (NSAIDs), and prescription of short-acting opioid narcotics (more than 85% of orthopedic surgeons report prescribing opioid narcotics after performing rotator cuff repairs) [8, 9].

The principal issue with this current pain relief strategy is the reliance on opioids, as the United States finds itself in the middle of the largest opioid epidemic in the world [10, 11]. Around two million Americans currently struggle with abuse of, or addiction to, opioid medication, leading to the United States accounting for 80% of opioid consumption worldwide [10]. Postoperative prescription and use of opioids in the United States is currently seven times higher than it is in Sweden and has been proven to be significantly higher than in Canada as well [11]. Specifically, orthopedic surgeons prescribe more opioid narcotics than any other medical specialty, accounting for 8.8% of all opioid dependence cases [10, 12]. Furthermore, a retrospective cohort study accounting for patients between 2001 and 2013 showed that opioid-naive surgical patients are significantly more likely to develop opioid dependence than matched comparison nonsurgical patients, particularly in patients over 50 [13]. Hence, there is a large desire in the field to reduce the prescription of opioids and a significant push for alternative pain relief modalities.

**Figure 1:**
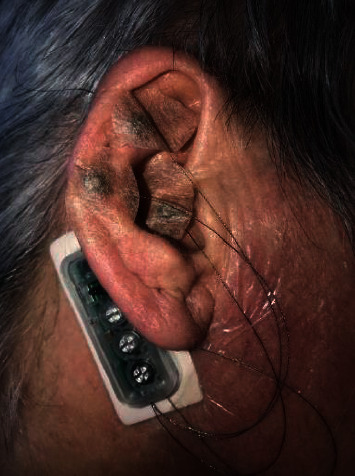
Image of the device with wires implanted in the patient's ear.

While some progress has been made in alternative pharmacologic modalities, such as the multimodal postoperative analgesic approach [14, 15], there is very limited literature available on peri and postoperative nonpharmacologic solutions. This case report adds necessary depth to the current literature surrounding nonpharmacologic alternatives by demonstrating the use of a nonpharmacologic auricular electrostimulation device, DyAnsys Primary Relief (FDA approved May 2018: K173861), as an analgesic after rotator cuff repair and biceps tenodesis operation. The Primary Relief device is a patented and FDA approved neurostimulation product that provides analgesia by conducting cranial electrostimulation through the passing of minute electrical currents to the brain via the auricular cranial nerves (Figure 1).

The objectives of the currently presented case report are to highlight one novel solution to a growing opioid epidemic in the field of orthopedic surgery, while simultaneously providing a base to the widely unexplored strategy of nonpharmacologic peri and acute postoperative analgesia. The authors believe that the successful completion of these objectives will prove to be a valuable addition to the current literature because, as the use of technology in orthopedic surgery rapidly escalates, it is only logical that nonpharmacologic, opioid-sparing modalities will quickly move towards the forefront of research in the field.

## 2. Case Presentation

The patient is a 73-year-old female who presented with a chief complaint of right shoulder pain and weakness for several months with failed nonsurgical management. Upon physical exam, she was identified to have weakness in the supraspinatus tendon and tendonitis of the long head of the biceps tendon. MRI confirmed a full-thickness tear of the supraspinatus from the footprint with one centimeter of retraction.

After reviewing all options, surgical and nonsurgical, the patient elected to undergo arthroscopy of the right shoulder. The operation performed was a rotator cuff repair and possible biceps tenodesis with bursectomy, with informed consent. The consent also included the application of a novel neurostimulation device to manage postoperative pain (DyAnsys Primary Relief). In this case, the device was used as an adjunct to traditional care, as outlined in the following sections.

## 3. Treatment

### 3.1. Therapeutic Intervention

Preoperatively, an interscalene block was administered by the anesthesiologist prior to general anesthesia. Arthroscopy of the right shoulder was then performed on the patient in a beach chair position with an arm positioner. Based on intraoperative findings, a rotator cuff repair was completed on the supraspinatus after bursectomy, followed by an open subpectoral biceps tenodesis. The Arthrex fixation system was utilized in this particular case for the cuff repair and the biceps. In addition to the main intervention, this patient also had a subacromial platelet-rich plasma (PRP) injection per patient request. There were no intraoperative complications and was otherwise routine for typical shoulder arthroscopies by the performing surgeon.

The patient recovered from surgery in PACU and was discharged home with a prescription of narcotics and a shoulder immobilizer.

A neurostimulation device was applied directly after the operation as an intended analgesic supplement.

### 3.2. Device Details

This neurostimulation device (DyAnsys Primary Relief) was applied directly after the operation (Figure 1). Before being administered, the stimulation parameters on the device were set to use a sweep frequency varying from 1.14, 2.28, 4.56, and 9.12 hertz every second, with pulse widths of 1000, 500, 250, and 125 microseconds, respectively; a biphasic pulse with an amplitude of approximately 3.5 volts was used. These settings were chosen because it is believed that low frequency variation accelerates the release of enkephalins to interact with mu and delta pain receptors [16–18].

Once the device had been programmed, auricular stimulation points were found using a nerve point locator to determine the point of lowest electrical resistance to a particular nerve (lowest electrical resistance simply meaning closest to the nerve); once located, these points were marked with a surgical marker for device implantation. The nerves stimulated in this case were the auricular branch of the vagus nerve (ABVN) in the concha, the auricular temporal nerve (ATN) in the vicinity of point zero on the ear, and the lesser occipital nerve (LON) in the vicinity of the shoulder point. These were chosen specifically for the operation being performed.

Next, the device was installed behind the patients' ear using a wearable product adhesive capable of lasting 15 days (the device battery lasts only 10 days). Once the device was stably adhered to the skin, the titanium needles at the end of titanium wires connected to the device were implanted, one at each of the previously marked nerve points and one ground needle inserted into the earlobe (Figure 1). Finally, tape was applied to ensure the needles stayed in place.

### 3.3. Outcome and Follow-Up

The patient presented to the office 2 weeks postoperatively with minimal pain and no narcotic use. The patient rated postoperative pain to be one out of ten beginning on postoperative day one through postoperative day ten when the neurostimulator was removed and replaced with a second identical device. The patient confirmed that although prescribed opioids and NSAIDs were offered, neither were used throughout the postoperative course. The patient felt no adverse effects of wearing the device throughout the day and the entire postoperative period. The patient did note, however, that there was a slight tingling sensation just prior to falling asleep, but this sensation was barely perceptible and not painful or uncomfortable.

At the two-week postoperative visit, the patient continued to report pain as being one out of ten, with no other issues. A physical exam showed tightness in the right shoulder, but this was determined to be treatable through standard physical therapy, and the general course of rehabilitation was maintained. However, at the patient's four-week postoperative clinic visit, she stated that she had fallen on her right shoulder which led to increased pain and weakness. Additional follow-up appointments were then scheduled for six and ten weeks from the date of surgery. At the ten-week follow-up, the patient reported, and the physical exam showed being back on an ideal rehabilitation timeline, only reporting low levels of pain with certain movements. By ten weeks, the patient also reported being back to usual daily activities, still without having taken any of the prescribed opioids or NSAIDs.

At the date of this article, it has been over one year since the operation and the patient continues to report that a full recovery has been made. All follow-up visits validate the findings in this report.

## 4. Discussion

Despite recent improvements in minimally invasive options for surgical rotator cuff repair, severe levels of postoperative pain can still be encountered [19, 20], causing prescribed opioids to be the mainstay for postoperative analgesia [8–10, 12, 19]. This reliance on opioids in orthopedic surgery [10, 12] is a major issue because of the current opioid epidemic in the United States [10, 11], as well as the significantly increased risk of opioid-naive patients to develop an opioid dependence postoperatively [13]. Even with the increased use of NSAIDs as an opioid-sparing postoperative analgesic, opioid consumption has only gone down 50% [19]. Furthermore, NSAIDs are a far from ideal modality as they have been linked to negative effects on bone healing, which can cause issues in biceps tenodesis operations [19]. Further adverse effects of NSAIDs have been described by Harirforoosh et al., such as including gastrointestinal, cardiovascular, and renal complications [21]. Hence, there is a highlighted need for nonpharmacologic analgesics in orthopedics. The primary nonpharmacologic pain relief device studied to date is transcutaneous electrical nerve stimulation (TENS) [22]. While TENS has been shown to be effective [22], the presented neurostimulator still adds significant depth to the literature because it provides analgesic efficacy 24 hours a day and does not limit the patients' daily activities while wearing the device. Oppositely, TENS can only be administered for a limited duration in a clinic (the treatment can be self-administered at home but only if the patient is able to purchase their own device meeting the surgeons' specifications), and the patient is unable to make significant movement during the treatment period [22].

Moreover, while prescribing these opioid-dominant analgesic and anti-inflammatory medications is a standard practice, the patient-directed nature of PRN medications can create a potential issue [23]. As rotator cuff repair is predominantly an outpatient procedure [24], patients are consistently offered unsupervised access to significant levels of opioids [25]. Although this is not a concern for most healthy, elective patients, there is a small percentage of undiagnosed addicts for whom this could be a high-risk circumstance [25, 26].

These factors highlight the value of a nonpharmacologic analgesic alternative, such as this novel auricular electrostimulation device. In the presented case, the patient was prescribed 5 mg/325 mg hydrocodone/acetaminophen (NORCO) with instructions. However, the patient did not take any of the prescribed narcotics. The patient was also instructed to use anti-inflammatory medications such as ibuprofen, yet the patient refrained from using these as well. While the lack of pain, and consequent lack of opioid use, in the immediate postoperative period can be explained by the interscalene nerve block [27], it is safe to attribute all analgesic efficacies after 72 hours to the neurostimulation device [27]. This suggests to the authors that the device exhibits a potential value in postoperative analgesia and is a promising step toward reducing the need for opioid analgesia in orthopedic patients.

That said, it is important to note the limitations of what is presented. One patient's outcome cannot be generalized to all patients. First, there are inadequate data for surgeons to safely rely on this or any other nonpharmacologic device in practice without also prescribing opioids as contingency modality. Multiple clinical trials would need to be run to show replication of the results seen in this case for device use without simultaneous opioid prescription to be a safe common practice. It is also worth considering the subacromial PRP injection utilized in this case as a potential confounding factor for postoperative pain levels. PRP has been shown in some studies to reduce pain after arthroscopic rotator cuff repair, though the overall evidence makes the extent of this effect unclear [9, 28]. Additionally, cost is a definite consideration for any device like this. It remains to be seen how much it would cost to implement the DyAnsys Primary Relief or a similar device on a large scale, compared to how much the current pharmacologic regimen costs.

Despite these potential limitations, the authors believe the use of auricular electrostimulation devices as peri and postoperative nonpharmacologic analgesic warrants further study, and the implementation of clinical trials to confirm the overwhelmingly positive results seen in this case should be undertaken immediately. This device also has the potential for massive implications into the future of surgical and nonsurgical pain management. Due to the limited published data on this nonpharmacologic device, it cannot be confirmed with certainty, but it is reasonable to assume that auricular electrostimulation and/or other forms of neurostimulation will be a large part of the future in pain management, theoretically expanding to use in major surgeries and chronic pain treatment.

## 5. Conclusion

Through the literature presented, it is apparent that a nonpharmacologic alternative to narcotics is needed to fulfill an unmet need of avoiding potential adverse events due to opioids and NSAID use after orthopedic surgery. The auricular electrostimulation device presented may provide one such solution, and further research should be undertaken to move towards clinical use of this or a similar device.

## Data Availability

No data were used to support this study.
